# Optimization of extraction process parameters of caffeic acid from microalgae by supercritical carbon dioxide green technology

**DOI:** 10.1186/s13065-022-00824-y

**Published:** 2022-05-13

**Authors:** Smritikana Pyne, Kishalay Paria

**Affiliations:** 1 Department of Food Technology and Biochemical Engineering, Jadavpur University, Kolkata, 700032 West Bengal India; 2 Oriental Institute of Science and Technology, Vidyasagar University, Midnapore, 721102 West Bengal India

**Keywords:** Microalgae, SC-CO_2_ extraction, Antioxidant, Caffeic acid, Preservative

## Abstract

**Purpose:**

In this study, the optimization of extraction process parameters of caffeic acid content from *Spirulina platensis* is performed by supercritical green technology.

**Methods:**

Especially, the optimization of supercritical carbon dioxide (SC-CO_2_) extraction parameters was carried out employing Box-Behnken design (BBD) and response surface methodology (RSM). Alongside, the three levels of extraction parameters i.e. extraction pressure, extraction time and temperature have been fixed. As a response, the caffeic acid content of the extracts was determined by HPLC. The statistical analysis (ANOVA) of developed mathematical models was used in the process.

**Results:**

The extract exhibited the highest content of caffeic acid as 72.11 µg/g of *dw* at the optimized extraction conditions of 360.08 bar pressure for 57.13 min extraction time at 38.31 °C temperature. Simultaneously this extract exhibited the highest content of total phenolic content (76.87 µg GAE/g *dw*), reducing power (2278 µg BHT/g *dw*), FRAP value (4.19 mM FeSO_4_ equivalent/g *dw*) and IC_50_ for DPPH activity (89.28 µg/mL).

**Conclusion:**

It has been also noted that supercritical fluid extract can significantly retard the growth of microorganisms in litchi beverage. Consequently, we can also predict that isolated SC-CO_2_ antioxidant containing fraction would have hopeful for foodstuff preservative.

## Introduction

Food has specific needs for providing balanced nutrition for human beings. The huge quantity of rotten foods is damped due to lack of proper preservation processe**s** in many developing and developed countries**.** Food preservation involves various food processing steps to sustain food quality including flavour at a desired level so that the highest benefits and nutritional values can be achieved. Some physicochemical preservation technologies like drying, chilling, freezing and pasteurization are used. The biologically derived antioxidant rich preservatives are capable of nutritional balance, flavour, texture as well as prevent loss of food quality. As of now, the production of supplemented food products in an escalating population is now a serious challenge in contrast with healthy diets and nutrition. Therefore, due to the occurrence of several bio-active components such as polyunsaturated fatty acids, β-carotene, antioxidants [1, 2], sulphated polysaccharides (anti-virals) and sterols (antimicrobials) [3], microalgae are considered as one of the supplemented food product for the enrichment of nutrition worth [4]. It is well documented that out of several bioactive compounds, caffeic acid (CA) is the most significant antioxidant rich phenolic component that widely distributed as hydroxylcinnamate and phenylpropanoid metabolites consisting of both phenolic and acrylic functional groups [5]. Again, caffeic acid (CA) acts as an antioxidant and anti-carcinogenic agent showing significant antibacterial activity [6]. In addition, it has antiviral activity as well as anti-inflammatory and immune-boosting ability [7]. Several extraction techniques have been performed to recover the phenolic components from microalgae. Subsequently, a variety of extraction factors may be required to achieve the maximum recovery of phenolic components [8]. The common conventional extraction method using hexane, ethanol and water was used for the collection of antioxidant rich molecules [9]. In case of long run various traditional procedures has many limitations, like; use of large quantities of toxic organic solvents, long extraction times, possess low selectivity, and/or low extraction yields. As of now to overcome these drawbacks, researchers were developed a new green technological approach, supercritical fluid (SCF) extraction for avoiding toxic organic solvents in food and pharmaceutical industry. It is evidence that SCF possesses physical properties intermediate between CO_2_ gas and a liquid above its critical point of temperature and pressure. Thereafter, the addition of a small amount of co-solvents can cause swelling of microalgae cells, facilitating the rapid mass transport of analytes from the matrix [10]. Strikingly, it was found that the extraction pressures and temperatures are set aside higher than the critical point of CO_2_ in supercritical carbon dioxide extraction (SC-CO_2_) [11]. It is widely documented that several researchers have been used SC-CO_2_ extraction technique for the purification of active components (as carotenoids and α-linolenic acid), oil and lipid from *Chlorella vulgaris* and *Scenedesmus obliquus* respectively [12, 13]. Extraction of caffeic acid from microalgae has been previously reported [14]. To our best information, there are no more previous studies on the antioxidant rich fraction of caffeic acid from *Spirulina platensis* using SC-CO_2_ extraction to date.

In such context, the present study has attempted to emphasize a new vista towards the work of optimizing the extraction parameters (temperature, pressure and time) using green extraction technology of SC-CO_2_. This fascinating observation describes of an SFE extract obtained under different extraction conditions having the best combination of caffeic acid content and phytochemical properties such as antioxidant, reducing power and total phenol. The strong antioxidant property of the SC-CO_2_ extract prompted us to investigate the antimicrobial properties of the same in preventing oxidative damage of beverage. Therefore this current research intended to obtain the optimized conditions of SC-CO_2_ extraction by Box-behnken Design (BBD) that would give up an antioxidant-rich extracts from *Spirulina platensis* for prospective application as preservative in food industries.

## Materials and methods

### Chemicals and reagents

It is considered as all chemicals, solvents, media used in this work were of analytical grade. The chemicals such as 2,2-diphenyl-1-picrylhydrazyl (DPPH), 2,4,6-tripyridyl-s-triazine (TPTZ), butylated hydroxy toluene (BHT), quercetin, gallic acid and caffeic acid were obtained from Sigma-Aldrich, Germany. Along with these chemicals, ethanol, Folin and Ciocateu’s phenol reagent, aluminium chloride, HPLC grade water, acetonitrile and acetic acid were purchased from Merck, Mumbai, India. The crystal of sodium carbonate anhydrous and sodium nitrite were from LOBA CHEMIE, Mumbai, India. Especially milli-Q system with a 0.22 μm filter paper (Millipore, Bedford, USA) was used to purify the mobile phase solution.

### Collection of microalgae sample

The microorganism *Spirulina platensis* var. lonor used in this study was collected from Antenna Green Trust, Madurai, Tamil Nadu, India. The samples were stored in screw capped amber colored plastic sealed container under dry and dark conditions.

### Analysis of proximate composition of microalgae biomass sample

The proximate analysis is used for the quantitative assessment of macromolecules of foods. It is confirmed that the proximate composition was assessed to find moisture, protein, ash, lipid, crude fiber and carbohydrate content of the microalgae biomass sample [15]. The moisture content was determined according to AOAC official method of 934.01by drying 5 g of sample in a hot air oven at 100–105 °C [16]. Along with, the crude protein was estimated by Kjeldahl protein units. The total amount of protein was determined by nitrogen percentage (%) × 6.25 [16] and total lipid content was measured by using hexane which described in AOAC official method of 920.39 [16]. Apart from that total ash was estimated by incineration of microalgae sample (5 g) at 550 °C during 2–3 h [16]. According to AOAC official method of 978.10, the crude fiber was estimated by subjecting 2 g of the de-fatted sample with 1.25% H_2_SO_4_ and 1.25% NaOH treatments as per AOAC, 2006 [16]. The total carbohydrate content of microalgae biomass was analyzed by difference. All proximate determinations were done in triplicate.

### Optimization of SC-CO_2_ conditions for extraction of caffeic acid from *Spirulina*

SC-CO_2_ extraction was accomplished by a Speed SFE System of Applied Separations, USA extraction unit according to the modified method of Dutta and Bhattacharjee, (2015) [17]. The system includes of a pump fixed with a cooling bath refrigerator at − 4 °C to make colder the pump. The fifty gram (50 g) of freeze dried *Spirulina* powder was weighed accurately and filled into a 100 mL of stainless steel vessel. In order to avoid leakage from the extraction vessel the cotton wool was to be put at both ends of the vessel. The compressed CO_2_ was added at flow rate of 2 L/min from the bottom of the extraction vessel through micro-metering valves. As a co-solvent, 100% ethanol was added at 1 mL/min from the bottom end of the extraction vessel.

In the present context, a BBD was employed for the optimization of SC-CO_2_ extraction conditions with three parameters of extraction pressure (200, 350 and 500 bar), temperature (30, 40 and 50 °C) and extraction time (static + dynamic) (60, 90 and 120 min) was employed in the present study. The dynamic time of 30 min was kept constant since no extract was collected after this time. After the extraction process all extracts were collected in glass vials. The extracts were gravimetrically weighed and dissolved in 96% ethanol. Then the extracts were successively stored in amber colored screw capped glass vials at 4 °C by purging a stream of nitrogen gas until further analyses. For comparative evaluation with conventional extraction methodology, solvent extraction was carried out with 10 g lyophilized biomass (5 g) in 50 mL of ethanol and was shaken at 200 rpm in a rotary shaker during 5 h at 30 °C. After that, the solution was centrifuged (SIGMA Laborzentrifugen 2–16 PK refrigerated centrifuge) at 13,500 g for 15 min and concentrated on a rotary vacuum evaporator (Supervac-R/180; Superfit Continental Pvt. Ltd., Mumbai, India) at 50 °C–60 °C. The extract was stored under conditions described above.

### Determination of caffeic acid content of *Spirulina* extracts by High performance liquid chromatography (HPLC)

The level of phenolic acid in extracts was quantified followed by the specific method with slight modifications [18]. The separation of phenolic acid was done on an Agilent ZORBAX SB C-18 column (150 × 4.6 mm, 5 μm) at 280 nm and 330 nm. The obtained each sample was dissolved in mobile phase solution (B) and filtered through a 0.22 μm polytetrafluorethylene syringe filter prior to HPLC injection at an injection volume of 20 μL into an Agilent 1300 series HPLC (Agilent Technologies Inc., Alpharetta, GA, USA). The eluent phase of water–acetic acid (95:5, v/v) (A) and methanol–acetonitrile–acetic acid (95:5:1, *v/v/v*) (B), starting from 0 to 40% B in 10 min, 40%–100% B in 10 min, 100% B in 5 min, and 100%–5% B in 5 min was used. The flow rate of solution was programmed at 1 mL/min. Therefore, the standard caffeic acid was solubilised in mobile phase B solution and concentration prepared at 100, 80, 60, 40, 20 µg/mL [19]. Every solution was injected three times using a needle. The mean peak areas were selected for the preparation of respective standard curve of caffeic acid and the results were expressed in µg/g of dry microalgae.

### Estimation of phytochemical properties of obtained *Spirulina* extracts

The total phenol content of the microalgae extracts was estimated by Folin and Ciocalteu reagent method [20] with slight modification. The total phenol contents were thus calculated as milligram gallic acid equivalent (GAE)/g *dw*. The reducing power of the extract was determined following the method of Ghosh et al. [21]. The reducing power was expressed as mg BHT equivalent/g *dw* from respective standard curve. Total flavonoids content of the extract was estimated by the AlCl_3_ method as described by Srivastava et al. (2012) [22]. The total flavonoid content was expressed as mg QE/g *dw*.

### Determination of antioxidant activities of extracts

The antioxidant activity of all extracts was determined by measuring the radical scavenging activity of DPPH [21] and expressed as IC_50_ values. The reducing ability was determined by Ferric reducing antioxidant power (FRAP) assay based on the reduction of a ferric-tripyridyltriazine complex to its ferrous colored form in the presence of antioxidants [23] and expressed as mM FeSO_4_ equivalent/g *dw* (from the standard curve prepared).

### Gas chromatography (GC–MS) analysis of the best SC-CO_2_ extract

The extract having the best combination of caffeic acid content and phytochemical properties was analyzed by GC–MS. GC–MS analysis of SC-CO_2_ extract were set according to the following method of Pantami et al. (2020) [24] with some modification. An Elmer GC Clarus Perkin 680 chromatograph (M/S, Perkin Elmer, MA, and USA) was used for the analyses. In particular, nitrogen gas was used at 1 mL/min as carrier. The column temperature was set firstly at 70 °C for 2 min, then increased to 150 °C at 25 °C/min, 200 °C at 3 °C/min, 260 °C at 8 °C/min and finally, settled at 260 °C for 1 min. After that, the best microalgae extract was diluted and automatically injected (1 µL) with 1:2 in split mode. As of now, injector and detector temperatures were put at 250 °C and 260 °C, respectively. The septum purge flow and gas saver mode were programmed at 3 mL/min and 20 mL/min. The MS source and MS Quad temperature were set at 230 °C and 150 °C with solvent delay by 12 min. The obtained peaks representing mass to charge ratio were compared with NIST mass spectrum library.

### Application of SC-CO_2_ extract of *Spirulina platensis* in litchi beverage

Ripe litchi was collected from Kharagpur local market. After washing with distilled water litchi juice were extracted by proper grinding and filtrating by muslin cloth. 2% sugar was added to prepared juice and heated on low flame. Then caffeic acid rich SC-CO_2_ algal extract (1 µg/mL) was added as preservative in litchi beverage. The treated juice was filled in glass bottle under sterilized condition. The glass bottles were shield immediately using a hand shieling machine for preventing contamination. Finally total two sets of studies were conducted by varying storage temperature viz. refrigerated (4 ± 2 °C) and ambient storage (room temperature) at specific time interval (0, 7, 15, 30, 45, 60 days).

### Sensory evaluation of litchi beverage

The litchi beverage preparations were sensorial evaluated by a panel of 10 trained judges comprising of research scholars of IIT Kharagpur. The sensory evaluation was selected by 9 point Hedonic Scale for different sensory attributes like colour, flavour, appearance and overall acceptability [25].

### Physical and chemical analysis

The litchi juice samples were taken from bottle at specific time interval and were analysed for total bacterial counts (TBC), total fungal count, total soluble/suspended solid, pH, acidity. TBC were estimated on nutrient agar media (Hi-media). After incubation at 37 °C for 24 h colony forming units (CFU) were counted. Fungal counts were estimated on potato dextrose agar (Hi media) after serial dilution of sample. After proper incubation at 28 °C for 72 h, CFU were counted. Total soluble solids (TSS) was determined using hand held refractometer (ERMA, Japan; 0–32) at room temperature. The pH of the litchi juice was determined with the help of digital pH meter (Systronics, Model 361). According to the method of Sadler and Murphy, (2010) [26] the titratable acidity was estimated.

### Statistical analysis

One-way analysis of variance (ANOVA) with Duncan's multiple range tests have been carried out to study the activities of extraction parameters such as temperature (^°^C) and pressure (bar) on the total yield of extract, total flavonoids, total phenolic content, antioxidant activity and reducing power of dried SC-CO_2_ extracts of microalgae. All statistical experiments were conducted using IBM SPSS Statistics 22 software (Statsoft, OK, USA) to verify the significance level at the p value of 0.05.

## Results and discussion

### Proximate analysis of microalgae biomass

The proximate analysis of *Spirulina* microalgae biomass showed that it contained 4.0 ± 0.36% moisture, 6.90 ± 0.53% fat, 61.8 ± 0.61% protein, 6.80 ± 0.40% crude fiber, 7.0 ± 0.20% ash, and 14.60 ± 1.08% carbohydrate (by difference) respectively on a dry weight basis.

### Characterization of obtained extract from *Spirulina platensis*

#### Total yield of microalgae extract

The amounts of extracts from microalgae *Spirulina platensis* under different extraction parameters by SC-CO_2_ extraction have been represented in Table 1. It was showed that, the total yield extract considerably increases (p = 0.002) with increasing temperature from 30 °C to 50 °C. It is also documented that antioxidant activity along with total phenol content and reducing power considerably reduced at higher temperature [27]. However, ANOVA analysis has revealed that the production of microalgae extract improved prominently (p = 0.02) with rising pressure from 200–350 bar. Thereafter the total yield extract increased significantly (p = 0.01) with increasing pressure up to 500 bar at constant temperature (40 °C).

**Table 1:** Phytochemical properties of obtained SFE extract by SC-CO_2_ extraction

Extraction pressure (bar)	Extraction temperature (°C)	Yield of extract (mg/g*dw*)	TPC (µg GAE/g *dw*)	Reducing power (µg BHT equivalent/g *dw*)	FRAP value (mM FeSO_4_ equivalent/g *dw*)	IC_50_ (µg/mL)
200	30.00	20.06 ± 0.75^a^	65.23 ± 3.44^c^	456.32 ± 12.01^ h^	1.21 ± 1.25^ h^	100.87 ± 4.25^ h^
200	40.00	15.98 ± 0.27^ g^	26.29 ± 1.57^i^	153.23 ± 7.10^ k^	0.4 ± 0.14^i^	180.10 ± 9.58^d^
200	40.00	8.74 ± 0.34^j^	20.99 ± 4.69^j^	199.68 ± 5.16^j^	3.22 ± 0.32^d^	140.45 ± 4.21^ g^
200	50.00	11.56 ± 0.29^d^	41.25 ± 2.21^f^	214.56 ± 10.09 ^i^	1.14 ± 0.47^ h^	100.24 ± 9.54^ h^
350	30.00	17.81 ± 0.98^f^	60.49 ± 3.05^d^	179.02 ± 10.72^ k^	1.27 ± 0.14^ h^	90.25 ± 2.14^i^
350	30.00	2.22 ± 0.37^i^	110.45 ± 3.58^a^	1124.25 ± 6.58^b^	2.35 ± 0.23^f^	105.89 ± 8.58^ h^
350	40.00	12.57 ± 0.22^d^	76.87 ± 1.89^b^	2278.46 ± 8.03^a^	4.19 ± 0.89^a^	89.28 ± 10.09^ h^
350	50.00	1.21 ± 0.72^ij^	16.08 ± 4.11^ k^	947.89 ± 4.81^e^	3.31 ± 0.81^c^	200.17 ± 7.14^c^
350	50.00	2.09 ± 0.07^i^	28.11 ± 1.79^i^	89.29 ± 7.04^ l^	3.29 ± 0.47^c^	204.83 ± 4.78^c^
500	30.00	10.22 ± 0.92^b^	42.56 ± 2.14^f^	700.13 ± 10.01^f^	4.09 ± 0.87^b^	211.52 ± 2.10^b^
500	40.00	4.80 ± 0.07^ h^	33.74 ± 1.89^ h^	579.73 ± 9.14^ g^	2.58 ± 0.56^e^	219.17 ± 6.45^a^
500	40.00	9.25 ± 0.22^e^	50.89 ± 3.58^e^	1068.29 ± 8.47^d^	2.25 ± 0.23^f^	157.22 ± 11.49^f^
500	50.00	4.57 ± 1.46^c^	38.24 ± 2.17^ g^	1155.21 ± 7.09^c^	2.17 ± 0.18^ g^	174.96 ± 7.28e

*dw*: Dry weight of sample; TPC: Total phenolic content (µg gallic acid equivalent (GAE)/g of dry weight (*dw*) of microalgae sample); IC_50_: IC_50_ value of DPPH radical scavenging activity

Different letters in a column indicate significant differences at p < 0.05 level

From the standard extraction theory of SC-CO_2_ it is noticed that the production of extract is also be influenced with intermolecular relations between the solute and co-solvent. The highest yield of extract (20.57 mg/g of dry microalgae) achieved by SC-CO_2_ extraction was at 350 bar, 40 °C. Since from the ANOVA analysis, it was also observed that together extraction pressure (p = 0.03) and temperature (p = 0.01) considerably can impact the yield of micro algae extract.

#### Phytochemical characterization of microalgae extracts

The amount of total phenol, FRAP value as well as reducing power activity of the algal extracts achieved in various SC-CO_2_ extraction parameters have been tabulated in Table 1. It was also set up that in the pressure of 350–500 bar of SC-CO_2_ extraction, the reducing power and total phenol content of the microalgae extracts increases (p < 0.02) along with rising temperature from 30–40 °C. Additionally the above phytochemical properties decreased in increasing (p = 0.06) the temperature to 50 °C.

It is common phenomenon that the solubility of solute and vapour pressure also increases during elevation of temperature. However, the effect of vapour pressure is significantly varied under higher pressure and temperature. The highest quantity of total phenolic content (110.45 μg GAE/g *dw*) was gained at 350 bar pressure 30 °C temperature and the reducing power activity (2278.46 μg BHT equivalent/g *dw*) was found at 350 bar, 40 °C. This was owing to the cross-over effect or retrograde phenomenon of temperature decreasing solubility in supercritical process [28].

#### Antioxidant activity of microalgae extracts

Under several conditions of SC-CO_2_ extraction, the obtained amounts of antioxidants from powdered algae have been represented in Table 1. It was displayed that the antioxidant activity of the extract increases (p = 0.00) in pressure of 300 bar–500 bar, with temperature of 30 °C–40 °C. The antioxidant activity reduced (p = 0.1) in increasing the temperature of 50 °C. Subsequently from ANOVA analysis it was also found that the antioxidant activity determined by both DPPH assays (p = 0.4563) and FRAP (p = 0.9801) does not considerably vary with the pressure. The maximum value of antioxidant activity measured by DPPH (IC_50_ = 89.28 µg/ml) and FRAP (4.19 mM FeSO_4_ equivalent/g *dw*) assays was achieved at 40 °C temperature and 350 bar pressure.

#### Caffeic acid content of microalgae extracts

The amount of caffeic acid obtained in conventional Soxhlet extraction was 54.01 ± 0.2 µg/g dry microalgae biomass using ethanol. This value was considered as the total content of caffeic acid in the freeze dried Spirulina microalgae biomass. The amount of caffeic acid of SC-CO_2_ extracts from microalgae under different extraction parameters is presented in Table 2. The maximum yield of caffeic acid (72.11 ± 1.05 µg/g *dw*) was achieved at 350 bar in 40 °C, during 60 min. The ANOVA table of regression model proved that yield of caffeic acid significantly increased (p = 0.01) with increasing temperature. Nonetheless, the yield of caffeic acid did not notably vary (p = 0.183) with increasing pressure from 200 to 350 bar. From the ANOVA analysis, it can be confirmed that two-level interaction of pressure–temperature did not significantly result (p = 0.92) but time–temperature have considerable effect (0.02) on the yield of caffeic acid content of microalgae extracts.

**Table 2:** Caffeic acid content of obtained SFE extract by SC-CO_2_ extraction

Total no. of runs	Pressure (bar)	Static time (min)	Temperature (^o^C)	Caffeic acid content (µg/g *dw*)
1	200	60	30.00	41.32 ± 2.31^c^
2	200	30	40.00	32.15 ± 3.56^e^
3	200	90	40.00	20.21 ± 3.12^i^
4	200	60	50.00	28.23 ± 1.84^ g^
5	350	30	30.00	36.38 ± 0.95^d^
6	350	90	30.00	22.54 ± 4.23^ h^
7	350	60	40.00	72.11 ± 1.05^a^
8	350	30	50.00	12.17 ± 4.78^j^
9	350	90	50.00	18.76 ± 7.10^ k^
10	500	60	30.00	44.23 ± 1.47^b^
11	500	30	40.00	40.21 ± 5.49^c^
12	500	90	40.00	22.45 ± 4.11^ h^
13	500	60	50.00	30.41 ± 7.08^f^

## Optimization of SC-CO_2_ extraction parameters for maximizing caffeic acid content using RSM

### Generation of 3D response curves

**Fig. 1:**
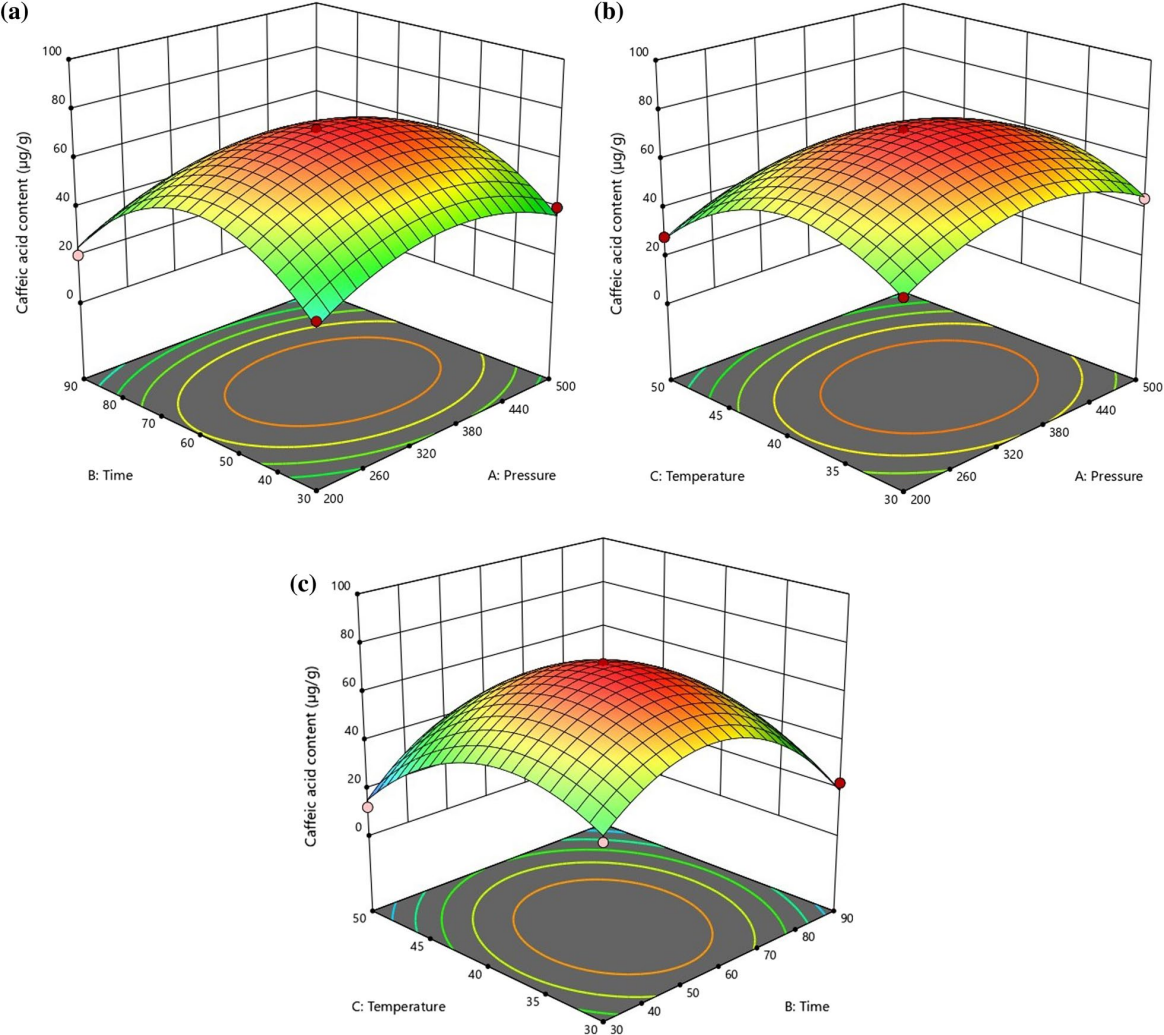
Response surface plot for caffeic acid content with respect to extraction pressure, temperature and extraction time and their mutual interactions

The effects of SC-CO_2_ extraction pressure, time and temperature on yield of caffeic acid were presented in Fig. 1a–c.

### Regression model development

The regression modelling was used for the response surfaces characterization. The response variable was fitted by a second order model in order to correlate the response variables to the independent variables. The general equation of the second degree polynomial equation is

1 $$ \begin{aligned} {\text{Y}}_{{\text{i}}} & = \beta_{{\text{o}}} + \beta_{{1}} {\text{X}}_{{1}} + \beta_{{2}} {\text{X}}_{{2}} + \beta_{{3}} {\text{X}}_{{3}} + \beta_{{{12}}} {\text{X}}_{{1}} {\text{X}}_{{2}} + \beta_{{{13}}} {\text{X}}_{{1}} {\text{X}}_{{3}} \\ & \quad + \beta_{{{23}}} {\text{X}}_{{2}} {\text{X}}_{{3}} + \beta_{{{11}}} {\text{X}}_{{1}}^{{2}} + \beta_{{{22}}} {\text{X}}_{{2}}^{{2}} + \beta_{{{33}}} {\text{X}}_{{3}}^{{2}} \\ \end{aligned} $$

where Y_i_ was set as response variable (caffeic acid content, β_o_ is the intercept; β_1,_ β_2_ and β_3_ are the linear coefficients; β_11,_ β_22_ and β_33_ are the quadratic coefficients; β_12,_ β_13_ and β_23_ are the interaction coefficient. The regression equation could be obtained by applying multiple regression analysis from Eq. (1). The expanded model includes linear, quadratic and cross-product terms as shown below (with intercept):

2 $$ \begin{aligned} {\text{Y}}_{{\text{i}}} & = + {36}.0{1 } - { 1}.{\text{79X}}_{{1}} + \, 0.{\text{564X}}_{{2}} - { 3}.{\text{43X}}_{{3}} + \, 0.00{2}0{\text{ X}}_{{1}} {\text{X}}_{{2}} + \, 0.0{\text{233X}}_{{1}} {\text{X}}_{{3}} \\ & \quad + \, 0.0{\text{17 X}}_{{2}} {\text{X}}_{{3}} + \, 0.0{\text{261X}}_{{1}}^{{2}} + {5}.0{\text{38E}} - 00{\text{3X}}_{{2}}^{{2}} + 0.{\text{215X}}_{{3}}^{{2}} \\ \end{aligned} $$

The effect of independent factors (pressure, temperature, and time) on the caffeic acid content (Y_i_) could be explained from Eq. (2). The residual analysis has been performed with the experimental data for the checking of the adequacy of the above regression model. The plot of predicted vs. observed values of caffeic acid content showed a close fit (r = 0.98) between the yield predicted by the design model and the experimental data. The ANOVA table (Table 3) was used to study the effect of each independent variable constructing a model that maximized the caffeic acid content of *Spirulina* extract. This analysis also provided values of the model term tested for adequacy and fitness. The significance of each variable was determined by its respective p-value and F-value at a specified level of confidence. The coefficient of determination (R^2^), the residual standard deviation (RSD) and the lack of fit were tested for the evaluation of goodness of fit and presented in ANOVA Table 3. The determination of coefficient (R^2^) was 0.98 and the p-value (> 0.05) for the lack of fit analysis was 0.152. The values of lack of fit were not significant, indicating that the model equations were adequate for predicting the caffeic acid content under any combinations of variable factors.

**Table 3:** ANOVA table for total yield and caffeic acid content of SFE extract

Source	Sum of squares	df	Mean square	F-value	Probability (P) > F	
*Yield of SFE extract*						
Model	469.62	9	52.18	24.24	0.0002	Significant
Lack of fit	11.71	3	3.90	4.64	0.086	Non-significant
Pure error	3.36	4	0.84			
Corrected total	484.69	16				
R^2^	0.97					
R^2^ adj	0.96					
*Caffeic acid content*						
Model	7961.70	9	884.63	64.95	< 0.0001	Significant
Lack of fit	66.54	3	22.18	3.08	0.15	Non-significant
Pure error	28.8	4	7.20			
Corrected total	8057.04	16				
R^2^	0.98					
R^2^ adj	0.97					

### Optimal processing conditions

The values of X_1_, X_2_, and X_3_ were determined to describe the most favourable processing conditions of SC-CO_2 `_extraction of caffeic acid from *Spirulina* microalgae. The regression equation were revealed with respect to X_1_, X_2_, and X_3_ values and set to zero. This was made by placing the second-order regression equation in matrix form. Thus the points acquired are recognized as the stationary point: X_1S_ = 360.08 bar, X_2S_ = 57.13 min and X_3S_ = 38.31 °C. The caffeic acid content of the extract at the stationary point were 74.17 µg/g of dry microalgae powder; whereas, the actual experimental values obtained were 72.11 µg/g of dry microalgae powder, suggesting a close fit of the model.

It was revealed from the above discussion that the obtained microalgae extract from *Spirulina platensis* by SC-CO_2_ extraction at 350 bar pressure and 40 °C temperature has the best prevalent combinational arrangement of total phenol content, antioxidant activity and reducing power.

### GC–MS analysis of optimized SC-CO_2_ extract

**Fig. 2:**
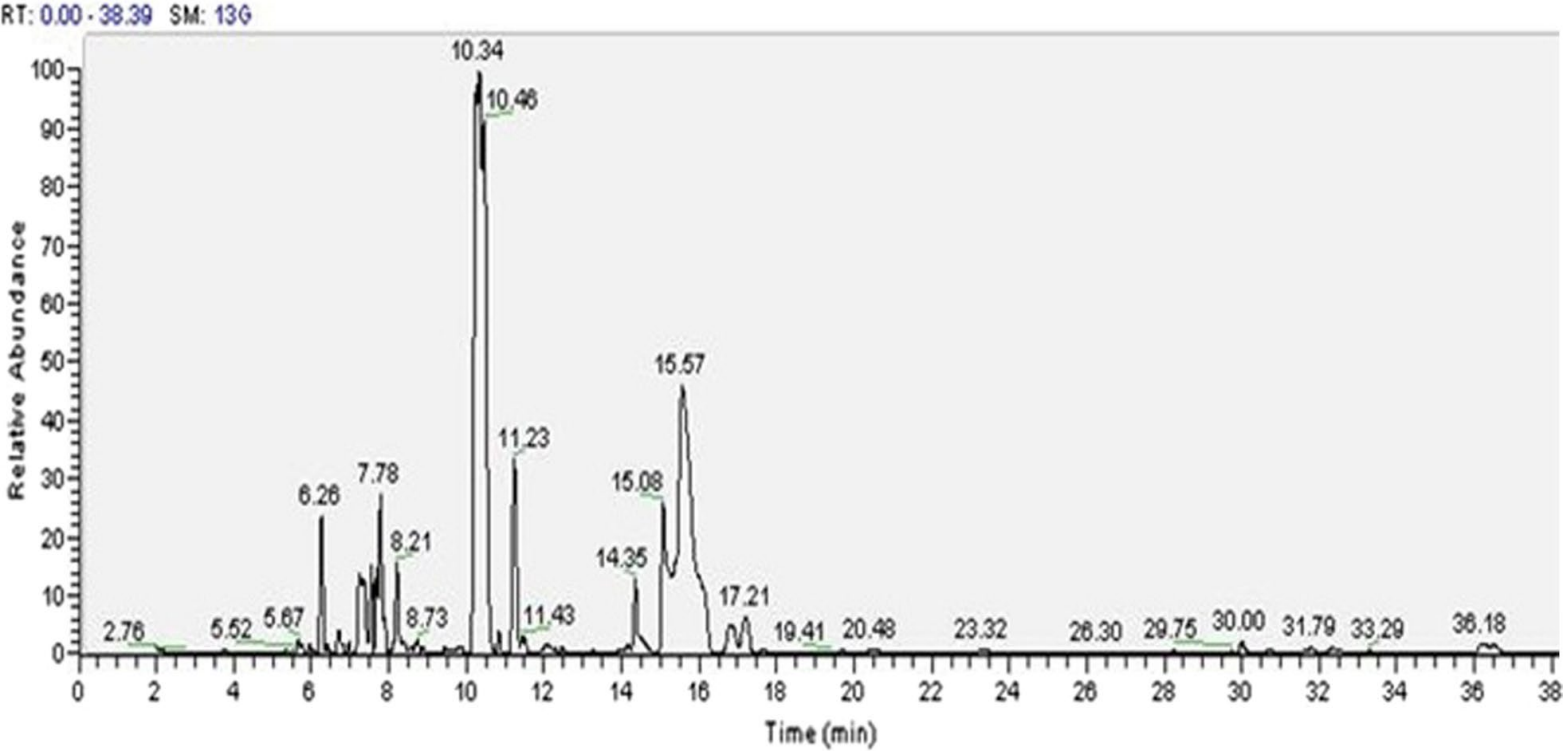
GC–MS analysis of optimized SFE extract

Fatty acids and triacylglycerol are the primary raw materials for lipids synthesis. Beside food industries, lipid has several kinds of application like detergent industries, bio-fuel or bio-energy production purpose etc. thereafter, it is important to assessment the fatty acid content of SFE extracts. The compounds in the SC-CO_2_ extract were recognized by GC–MS analysis (Fig. 2) and have been provided in Table 4. It is observed that palmitic acid is the primary compound of the antioxidant rich algal extract. Besides that benzenepentanoic acid methyl ester, decane 3 bromo, tetradecanoic acid methyl ester, propanoic acid methylethyl ester, linoleic acid, stearic acid and eicosapentaenoic acid are the other extracted components, all of which have appreciable nutraceutical properties.

**Table 4:** GC–MS analysis of optimized SC-CO_2_ extract

Serial no	R.T. (min)	Peak area (%)	Compounds identified
1	6.26	3.17	2-hexyl-1-octanol
2	6.71	0.72	5-phenyl undecane
3	7.24	3.81	2-methyl,7 octadecyne
4	7.78	4.92	Irididiol
5	8.21	2.22	4-tetradecyne
6	10.34	42.94	Pentadecanoic acid
7	11.23	6.18	Palmitic acid
8	13.26	0.06	Oxalic acid
9	14.35	2.38	Oleic acid
10	15.08	6.25	Pentadecatriene
11	15.56	15.37	2,7-Methanonaphthalene-3 methanol
12	17.21	1.81	Linoleic acid

### Sensory studies of litchi beverages

#### Changes in quality of litchi beverages

**Fig. 3:**
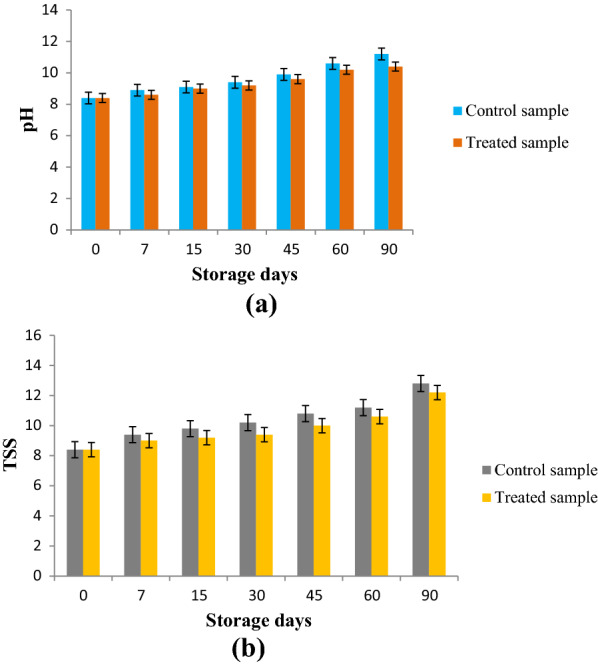
Change in pH of the litchi juice under refrigerated (**a**) and ambient storage (**b**)

**Fig. 4:**
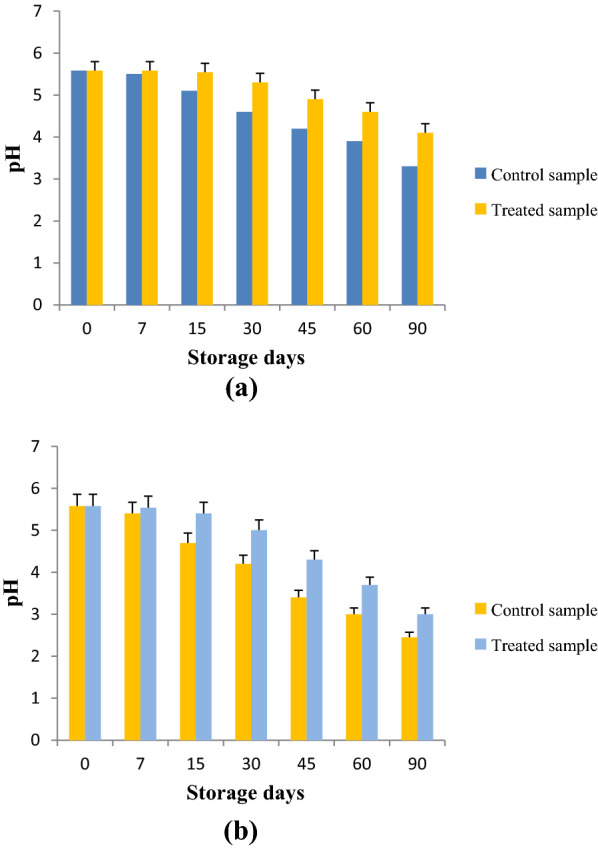
Change in TSS of the litchi juice under refrigerated (**a**) and ambient storage (**b**) condition

The different physico-chemical and biological quality parameters of litchi juice using SC-CO_2_ extract under refrigerated and ambient temperature were considered. The good quality of food product was first determined by pH. Fig. 3 showed the changes of pH values in the litchi beverage samples during storage. It showed that, during the increase of storage period along with temperature, pH decreased whereas the acidity and TSS of beverage were increased. The data revealed that at refrigerated storage on day 30^th^, pH decreased. In contrast TSS of the beverage was observed to be higher in ambient storage as compared to refrigerated storage (Fig. 4). On day 30^th^ acidity of juice increased at refrigerated storage.

#### Microbial count of beverages

At ambient storage more microbial growth was observed as compared to storage at refrigerated condition. It was found that total bacterial and fungal counts are less in SC-CO_2_ extract treated sample than untreated once (Tables 5, 6).

**Table 5:** Total bacterial count CFU/ml on Nutrient agar medium

Days	Control sample at 4 °C	Treated sample at 4 °C (SFE extract added)	Control sample at 28 °C	Treated sample at 28 °C (SFE extract added)
0	Nil	Nil	Nil	Nil
7	Nil	Nil	350,000 + -10	Nil
15	40,000 + -10	Nil	500,000 + -10	Nil
30	140,000 + -10	Nil	900,000 + _10	80,000 + _10
45	300,000 + -10	Nil	uncountable	1,250,000 + _10
60	uncountable	Nil	uncountable	uncountable

**Table 6:** Total fungal count CFU/ml Potato Dextrose Agar (PDA) Medium

Days	Control sample at 4 °C	Treated sample at 4 °C (SFE extract added)	Control sample at 28 °C	Treated sample at 28 °C (SFE extract added)
0	Nil	Nil	Nil	Nil
7	Nil	Nil	20,000 + -10	Nil
15	Nil	Nil	50,000 + -10	Nil
30	10,000 + _10	Nil	100,000 + -10	Nil
45	40,000 + -10	Nil	200,000 + -10	18,000 + -10
60	90,000 + -10	Nil	300,000 + -10	60,000 + -10

#### Shelf life analysis of beverages

The suitability of food products during refrigerated storage is related with the variations in their intriguing sensory properties. As assessed by the sensory panel, the scores for colour, appearance, taste, odor and overall acceptability of the litchi beverage decreased significantly during the long storage time. The results represented that the beverage fortified with SC-CO_2_ extract have significantly much better sensory scores. The storage interval of the treated and untreated beverages was judged on the basis of sensory quality. The sensory score of 6 and above on 9 point Hedonic scale rating was used as a threshold for estimating the shelf life of beverage [29]. The litchi beverage sample with SC-CO_2_ extract earned relatively better sensory score than untreated sample on 30 days of storage period during refrigerated condition and 15 days during ambient storage. The analysis of obtained result showed that storage condition and treatment had a significant effect on over all acceptability of litchi beverage.

### Practical application

Food biotechnology has been used for food industries that provide nutritional quality in a cost effective manner. This research has focused on exploration of natural preservatives in the human community due to no side effects as chemical preservatives. Our knowledge about the application of *Spirulina* derived material in the food preparation and preservation sector is still in its infancy. In view of all propositions the application of *Spirulina* has emerged as an efficient option for food preparation but many challenges and constraints with regard to its practical applications on a large commercial scale still prevail. The following points are needed to be addressed to get the adequate benefit of the *Spirulina* for practical applications in food industries.

1. Chemical preservatives in the food sector are a serious threat to human health. The feasibility of *Spirulina* is cost-effective and efficiently explored.
2. The mechanism of preservative has not yet been fully understood and demands more generation of transparent research outcomes in the preservative of the bactericidal and fungicidal mechanism.
3. The use of *Spirulina* derived nanomaterial may become an alternative tool for food preservation.

## Concluding remarks

The validated facts are that *Spirulina platensis* is a useful natural gift having numerous potential applications in bio-resources based food and feed industries. *Spirulina* microalgae have divers group of metabolites including carotenoids, amino acid, fatty acid, antioxidant, caffeic acid that enhance the nutritional value of human food and feed. A few examinations indicated that, most of the rising methods for getting better recovery of bioactive components still necessitate further research to get to industrial level. For that reason, in order to develop large scale and energy efficient production of bioactive compounds from *Spirulina*, the reliable extraction method i.e. SC-CO_2_ is studied. Unmistakably, caffeic acid has numerous antioxidant properties which might decrease the risk of microbial infection on various foods. As discuss in this research, the caffeic acid content depends upon a variety of factors including change of extraction temperature, pressure and time. From the above discussion we have inferred two main issues- First, the maximum total yield of algal extract with best combination of phytochemical properties from *Spirulina platensis* microalgae by SC-CO_2_ extraction were obtained at 350 bar pressure and 40 °C temperature during 60 min extraction time. Secondly, caffeic acid has significant impact on food preservation.

## Future perspectives

Presently SFE extract of *Spirulina* is used as an alternative to chemical preservatives. Different chemicals such as benzoates, potassium sorbate, butylated hydroxyanisole, sodium bisulfite are introduced as preservative agents to reduce microbial growth of packaged food. Although SFE extract of *Spirulina* is too much expensive but it is recommendable in so many valuable food products like space food, fermented food. In this field, the protective role of *Spirulina platensis* as antioxidant has already been documented. Determining the right category of *Spirulina* strain, the extraction of caffeic acid in specified amount and its utility should be studied in future. We trust that this article will be useful in shaping the future course of food preservation based research.

## Data Availability

All data and materials analyzed during the current study are included in this manuscript.

## Abbreviations

SC-CO_2_: Supercritical carbon dioxide
HPLC: High performance liquid chromatography
ANOVA: Analysis of variance
GAE: Gallic acid equivalent
CA: Caffeic acid
DPPH: 2,2-Diphenyl-1-picrylhydrazyl
BHT: Butylated hydroxytoluene
SFE: Supercritical fluid extract
